# Commentary: Rapalink-1 Increased Infarct Size in Early Cerebral Ischemia–Reperfusion With Increased Blood–Brain Barrier Disruption

**DOI:** 10.3389/fphys.2021.761556

**Published:** 2021-09-22

**Authors:** Daniel J. Beard, Gina Hadley, Brad A. Sutherland, Alastair M. Buchan

**Affiliations:** ^1^School of Biomedical Sciences and Pharmacy, University of Newcastle, Newcastle, NSW, Australia; ^2^Laboratory of Cerebral Ischaemia, Radcliffe Department of Medicine, University of Oxford, Oxford, United Kingdom; ^3^Tasmanian School of Medicine, College of Health and Medicine, University of Tasmania, Hobart, TAS, Australia

**Keywords:** stroke, mTOR–mammalian target of rapamycin, neuroprotection, rapamycin, Rapalink-1

## Introduction

We read with great interest the article by Chi et al. investigating the effect of inhibiting mammalian target of rapamycin (mTOR) with a third generation mTOR inhibitor, Rapalink-1, in an experimental model of stroke (Chi et al., 2021). Rapalink-1 increased stroke size which was associated with reduced activity of both mTORC1 and mTORC2. In this commentary we wish to use insights provided by Chi et al. to clarify the emerging inconsistencies in the literature surrounding the brain cytoprotective potential of mTOR inhibition in stroke, and the different roles of mTORC1 and mTORC2. We will highlight lessons learned about optimal dosing of rapamycin in experimental stroke in an attempt to inform dosing of newer generation mTOR inhibitors such as Rapalink-1, to achieve brain cytoprotection.

## mTOR, A Double Edge Sword in Brain Cytoprotection Following Ischemia

mTOR exists in two multiprotein complexes: mTORC1 and mTORC2. mTORC1 is an energy sensor within cells. If cellular energy is abundant mTOR is activated leading to protein synthesis and cell proliferation through activation of ribosomal protein S6 kinase (p70S6K), eukaryotic initiation factor 4E-binding protein 1 (4E-BP1), and autophagy inhibition (Wang et al., 2014). Stresses such as depleted energy supply induce both TSC1 (hamartin) and TSC2 (tuberin) complex activity to inhibit mTORC1 (Deyoung et al., 2008). The role of mTORC2 is not as well-established. Studies have shown it plays a role in maintaining cytoskeletal integrity and cell survival (Shin et al., 2015).

mTOR plays a central role in cell death following cerebral ischemia (Chong et al., 2013). We have shown during global ischemia, resistant cells within the hippocampus (CA3) can up-regulate hamartin leading to mTORC1 inhibition, increasing productive autophagy to promote cell survival (Papadakis et al., 2013). mTORC1 can be inhibited pharmacologically using the mTORC1 inhibitor, rapamycin, which has been proposed as a potential therapy for stroke. Studies of rapamycin in experimental stroke produced mixed results (Hadley et al., 2019b), with some studies showing rapamycin reduced infarct volume (Buckley et al., 2014) and others showing increased infarct volume (Chi et al., 2016a). Our subsequent systematic review and meta-analysis demonstrated that rapamycin has an overall protective effect in animal models of stroke with no evidence of publication bias (Beard et al., 2019). We found a large proportion of the variability in outcome with rapamycin treatment is due to the dose of rapamycin, with the lower dose (<2 mg/kg) being most protective and higher doses (20 mg/kg) increasing infarct volume. This provides evidence that dosing is paramount when considering rapamycin in stroke.

Chi et al. used the highest doses of rapamycin (2 × 20 mg/kg pre-treatment), providing the first clues for the role of mTORC2 in cerebral ischemia. Increased infarct volumes in these studies were associated with inhibition of mTORC2 (Chi et al., 2016a,b). This is a known off-target effect of prolonged or high dose rapamycin treatment, as it prevents association of mTOR with mTORC2 components rictor and SIN1 (Sarbassov et al., 2006). We have also shown that the dual mTORC1/2 inhibitor AZD2014 increases cell death in cortical neurons during oxygen glucose deprivation (Hadley et al., 2019a). This effect is likely due to the inability of mTORC2 to phosphorylate and activate the AKT pro-survival pathway (Shin et al., 2015). Further studies by Chi et al. support this hypothesis, showing that selective inhibition of the mTORC1 pathway via inhibiting p70S6K with PF-4708671, reduces infarct volume in an experimental stroke model (Chi et al., 2019) and activating AKT with SC79 also reduces infarct volume (Liu et al., 2018). The conflation of these recent findings suggests that during ischemia, acutely inhibiting mTORC1 while maintaining or enhancing mTORC2 activity is the optimal strategy for brain cryoprotection.

## mTOR Inhibitors in Ischemic Stroke, Less May Be More

Rapamycin/rapalogues are already clinically approved to prevent organ transplant rejection (Knight and Kahan, 2006), cancers (Fagone et al., 2013), and cardiovascular disease (Sousa et al., 2001). They are an attractive therapy to be repurposed for stroke, as they have already passed early phases of development and clinical testing. Approval for this new indication could therefore take less time at a substantially reduced cost (Ashburn and Thor, 2004). Chi et al. provide the first experimental stroke study of the mTOR inhibitor, Rapalink-1 (Chi et al., 2021). Rapalink-1 combines rapamycin with the mTOR kinase inhibitor, MLN0128 (molecular weight of Rapalink-1= 1,784 g/mol vs. rapamycin = 914 g/mol), with the goal of leveraging the high affinity of rapamycin for mTORC1 (via FK506 Binding Protein 12) to selectively deliver MLN0128 to the ATP-site of the mTORC1 complex (Rodrik-Outmezguine et al., 2016). Early studies indicated Rapalink-1 was a more potent mTORC1 inhibitor than rapamycin, inhibiting growth and suppressing S6 and 4EBP1 phosphorylation in a glioblastoma cell line and in the brain, at doses as low as 1.56 nM and 0.4 mg/kg, respectively (Fan et al., 2017). This is compared to rapamycin which only suppressed S6 phosphorylation at ≥6.25 nM and 4 mg/kg, respectively. Only higher concentrations of Rapalink-1 (≥6.25 nM) were found to suppress mTORC2/AKT *in vitro*, compared to rapamycin (>200 nM) (Fan et al., 2017). Given the increased potency of Rapalink-1, it would be expected that doses of Rapalink-1 needed for brain cytoprotection in stroke would be much lower than rapamycin (optimal dose for rapamycin <2 mg/kg). Chi et al. used 2 mg/kg of Rapalink-1, which was associated with mTORC1 and mTORC2 inhibition and increased infarct volume (Chi et al., 2021). These findings further highlight the importance of mTORC2 signaling for cell survival during ischemia and suggest that although the dose of Rapalink-1 used is not too dissimilar to cytoprotective doses of rapamycin, the increased potency of Rapalink-1 may have resulted in mTORC2 inhibition at a lower dose.

## Discussion

If we are to use rapamycin and Rapalink-1 to treat stroke, dosing is key. Although our meta-analysis suggests that lower doses of rapamycin are brain cytoprotective, the mechanism is unclear. This could be mediated by lower dose rapamycin producing mTORC1 inhibition while sparing mTORC2 activity. A murine study found that 2 mg/kg of rapamycin can inhibit S6 phosphorylation (mTORC1) without reducing AKT activity (mTORC2) in skeletal muscle (Arriola Apelo et al., 2016). Except for Chi et al. no previous stroke study investigating rapamycin or Rapalink-1 have measured mTORC1/2 activity in the brain. Future studies of rapamycin and Rapalink-1 in stroke must conduct dose-response studies to identify the optimal dose that inhibits mTORC1 while maintaining mTORC2 activity.

## Author Contributions

DB wrote the first draft of the manuscript. GH, BS, and AB contributed to manuscript revision and approved the submitted version. All authors contributed to the article and approved the submitted version.

## Conflict of Interest

AB is a co-founder of Brainomix, a company that develops electronic ASPECTS (e-ASPECTS), an automated method to evaluate ASPECTS in stroke patients. The remaining authors declare that the research was conducted in the absence of any commercial or financial relationships that could be construed as a potential conflict of interest.

## Publisher's Note

All claims expressed in this article are solely those of the authors and do not necessarily represent those of their affiliated organizations, or those of the publisher, the editors and the reviewers. Any product that may be evaluated in this article, or claim that may be made by its manufacturer, is not guaranteed or endorsed by the publisher.
